# Upregulation of miR-941 in Circulating CD14+ Monocytes Enhances Osteoclast Activation via WNT16 Inhibition in Patients with Psoriatic Arthritis

**DOI:** 10.3390/ijms21124301

**Published:** 2020-06-17

**Authors:** Shang-Hung Lin, Ji-Chen Ho, Sung-Chou Li, Yu-Wen Cheng, Yi-Chien Yang, Jia-Feng Chen, Chung-Yuan Hsu, Toshiaki Nakano, Feng-Sheng Wang, Ming-Yu Yang, Chih-Hung Lee, Chang-Chun Hsiao

**Affiliations:** 1 Department of Dermatology, Kaohsiung Chang Gung Memorial Hospital and Chang Gung University College of Medicine, Kaohsiung 83301, Taiwan; hong51@cgmh.org.tw (S.-H.L.); jichenho@cgmh.org.tw (J.-C.H.); yuwen@cgmh.org.tw (Y.-W.C.); yichienyang@gmail.com (Y.-C.Y.); 2 Graduate Institute of Clinical Medical Sciences, College of Medicine, Chang Gung University, Taoyuan 33302, Taiwan; toshi.nakano@msa.hinet.net (T.N.); yangmy@mail.cgu.edu.tw (M.-Y.Y.); 3 Chang Gung University of Science and Technology—Chiayi Campus, Chiayi 61363, Taiwan; 4 Genomics and Proteomics Core Laboratory, Kaohsiung Chang Gung Memorial Hospital, Chang Gung University College of Medicine, Kaohsiung 83301, Taiwan; raymond.pinus@gmail.com; 5 Division of Rheumatology, Allergy and Immunology, Department of Internal Medicine, Kaohsiung Chang Gung Memorial Hospital and Chang Gung University College of Medicine, Kaohsiung 83301, Taiwan; uporchid@cgmh.org.tw (J.-F.C.); chungyuango@gmail.com (C.-Y.H.); 6 Department of Surgery, Kaohsiung Chang Gung Memorial Hospital, Kaohsiung 83301, Taiwan; 7 Department of Medical Research, Kaohsiung Chang Gung Memorial Hospital and Chang Gung University College of Medicine, Kaohsiung 83301, Taiwan; wangfs@ms33.hinet.net; 8 Department of Otolaryngology, Kaohsiung Chang Gung Memorial Hospital and Chang Gung University College of Medicine, Kaohsiung 83301, Taiwan; 9 Center for Shockwave Medicine and Tissue Engineering, Kaohsiung Chang Gung Memorial Hospital, Kaohsiung 83301, Taiwan

**Keywords:** psoriatic arthritis, osteoclastogenesis, miR-941, WNT16

## Abstract

Psoriatic arthritis (PsA) is a destructive joint disease mediated by osteoclasts. MicroRNAs (miRNAs) regulate several important pathways in osteoclastogenesis. We profiled the expression of miRNAs in CD14+ monocytes from PsA patients and investigated how candidate microRNAs regulate the pathophysiology in osteoclastogenesis. The RNA from circulatory CD14+ monocytes was isolated from PsA patients, psoriasis patients without arthritis (PsO), and healthy controls (HCs). The miRNAs were initially profiled by next-generation sequencing (NGS). The candidate miRNAs revealed by NGS were validated by PCR in 40 PsA patients, 40 PsO patients, and 40 HCs. The osteoclast differentiation and its functional resorption activity were measured with or without RNA interference against the candidate miRNA. The microRNA-941 was selectively upregulated in CD14+ monocytes from PsA patients. Osteoclast development and resorption ability were increased in CD14+ monocytes from PsA patients. Inhibition of miR-941 abrogated the osteoclast development and function while increased the expression of WNT16. After successful treatment, the increased miR-941 expression in CD14+ monocytes from PsA patients was revoked. The expression of miR-941 in CD14+ monocytes is associated with PsA disease activity. MiR-941 enhances osteoclastogenesis in PsA via WNT16 repression. The miR-941 could be a potential biomarker and treatment target for PsA.

## 1. Introduction

Psoriatic arthritis (PsA) is a chronic and indolent inflammatory disease involving progressive arthropathy in approximately 30% of patients with psoriasis. Skin manifestations usually precede the onset of PsA by an average of 10 years. [1]. The diagnosis of PsA is based on the recognition of clinical and imaging features [2]. The most widely used diagnostic criteria for PsA is the Classification Criteria for Psoriatic Arthritis (CASPAR) [3]. PsA is easily overlooked and missed with incorrect diagnosis or messed up with delayed diagnosis, both of which could lead to poor radiographic and devastating functional outcome [4]. In fact, Haroon et al. reported that a diagnostic delay of more than six months contributes to poor radiographic and functional outcome in PsA [5]. 

PsA is featured with bone erosions mediated by activated osteoclasts. Osteoclasts, the multinucleated giant cells with a monocyte/macrophage lineage, are the main cells responsible for bone resorption [6,7]. It was reported that numbers of osteoclast precursors are increased in PsA patients as compared with those from healthy controls [8]. Consistently, we have demonstrated that circulating CD14+ monocytes from patients with PsA have active osteoclastogenesis and active resorption activity. 

MicroRNAs (miRNAs) are a class of small noncoding RNAs that negatively regulate the expression of protein-coding genes. Emerging evidence suggests that miRNA-mediated regulation represents a fundamental layer of epigenetic control over diverse physiological and pathological processes [9,10]. 

With a hypothesis-driven approach, we previously investigated the role of three common osteoclast activation microRNAs (miR-146a/b and miR-155) in CD14+ monocytes in PsA. We demonstrated miR-146a-5p in CD14+ monocytes from PsA patients correlates with its disease severity in vivo and active bone resorption in vitro [11]. However, in that study, only three microRNAs are investigated in osteoclasts of PsA patients. A general expression profile of various miRNAs in PsA is required to investigate whether there were other important miRNAs critical in the pathogenesis of PsA. 

Next-generation sequencing (NGS) provides a high-throughput sequencing platform that performs much better than the traditional Sanger sequencing. NGS facilitates the discovery of genes and regulatory elements associated with disease [12] so that we could determine miRNA expression profile of the pooled RNA libraries from osteoclasts of PsA patients, psoriasis patients without arthritis (PsO), and healthy controls (HC). We adopted MiSeq platform (Illumina) for large scale profiling. The RNA libraries are first prepared with TruSeq Small RNA Sample Preparation protocol (Illumina) followed by sequencing with MiSeq platform. The generated NGS data is further analyzed with miRSeq for evaluating sequencing quality and determining miRNA expression profile.

Circulating monocytes in the blood are appropriate and readily accessible for bone-related studies and are a good source of osteoclast precursors to study [13,14]. Therefore, in this study, we utilized circulating monocytes to investigate the functional activation osteoclasts in individual subjects. In addition, the overall expression of miRNAs from osteoclast precursors, the CD14+ monocytes, has not been profiled independently. The present study aimed to identify whether specific miRNAs (through NGS) from CD14+ monocytes could serve as diagnostic biomarkers and treatment targets for PsA (through clinical subject categories). This study also addressed the mechanisms by which specific miRNAs contribute to active osteoclastogenesis and functional activity in PsA (through RNA interference and bone resorption assay).

## 2. Results

### 2.1. Subject Demographics

Forty patients with PsA (Female/Male: 12/28, average age: 47.6 years old), 40 PsO patients (Female/Male: 9/31, average: 43.8 years old), and 40 HCs (Female/male: 11/29, average age: 44.1 years old) were recruited (Table 1). Most PsA patients had severe psoriasis (average PASI of 14.2), and all of them had peripheral arthritis, including 35% with axial arthritis, 35% with dactylitis, and 45% with enthesitis.

### 2.2. Upregulation of miR-941 in CD14+ Monocytes from PsA Patients by qRT-PCR, with Support Vector Machine Learning, Identified miR-941 as an Early Predictor for PsA

**Figure 1:**
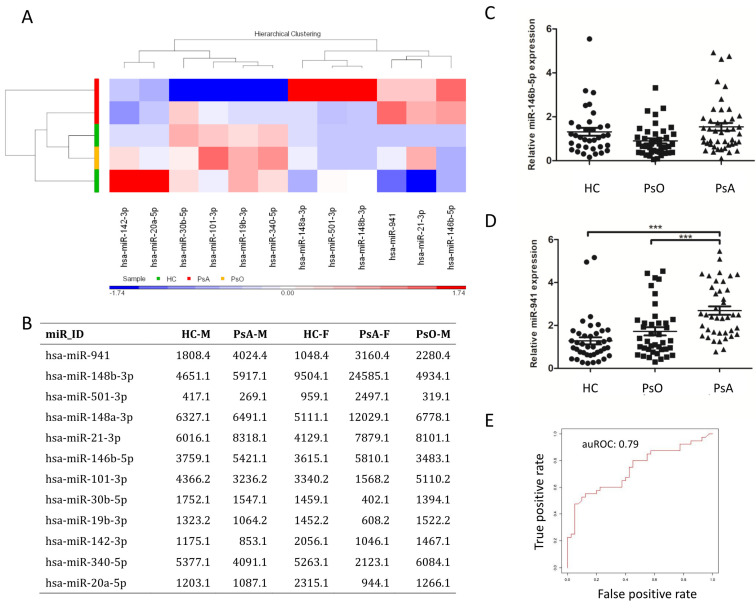
High expression of miR-941 in CD14+ monocytes from PsA patients. (**A**) The RNA samples were analyzed from (1) the healthy control group (HC), including two pooled samples from two males and one female, respectively; (2) the psoriasis group (PsO), including one pooled sample from two males; and (3) the psoriasis arthritis group (PsA), including two samples from two males and one female, respectively. MicroRNAs (miRNAs) with transcript per million (TPM) values more than 1000 in at least one sample and with average expression ratio (normal to disease or disease to normal) more than 1.5 are plotted on the heat map. (**B**) The microRNA expression level was presented in the unit of TPM. (**C**,**D**) The expression levels of miR-146b-5p and miR-941 were measured in HCs (*n* = 40), PsO patients (*n* = 40) and PsA patients (*n* = 40) by quantitative real-time reverse transcription polymerase chain reaction (qRT-PCR). Patients with PsA showed increased expression of miR-941 in CD14+ monocytes. (**E**) We set training model from miR-941 expression of CD14+ monocytes from patients with PsA, PsO, and HCs using a Support Vector Machine (SVM) learning algorithm. To distinguish PsA from PsO and HC, the value of auROC was 0.79, indicating that miR-941 expression can distinguish PsA patients from PsO and HCs with high accuracy. *** *p* < 0.001.

In order to screen specific miRNA expressions in patients with PsA, we first profiled the miRNA expression from two pooled samples from HCs (from two males and one female, respectively), two pooled samples from PsA patients (from two males and one female, respectively) and one pooled sample from PsO patients (from two males). We collected approximately 32.0 million raw reads in total, and each sample yielded 6.4 million reads on average. Twelve miRNAs with transcripts per million (TPM) values higher than 1000 in at least one sample and with average expression ratios relative to HCs > 1.5 were identified. The overall expression levels of these 12 miRNAs from PsO, PsA, and HC monocytes are presented in Figure 1A,B. Notably, expression of miR-941 and miR-146b-5p were 1.5-fold greater in the PsA group compared to that in both HC and PsO group. To validate the results revealed by NGS, the expression of miR-941 and miR-146b-5p were measured by quantitative real-time reverse transcription polymerase chain reaction (qRT-PCR) in CD14+ monocytes from 40 PsA, 40 PsO, and 40 HC subjects. While expression level of miR-146b-5p was similar among groups (Figure 1C), miR-941 expression was significantly greater in PsA patients than that in both PsO patients and HCs (Figure 1D). MiR-941 expression was significantly higher in CD14+ monocytes derived from PsA patients compared to PsO (ORs: 2.87 (95% CI: 1.62–5.06), *p* < 0.001) or HCs (ORs: 3.85 (95% CI: 2.12–6.98), *p* < 0.001). Therefore, miR-941 could be a potential biomarker able to identify PsA patients from PsO or HCs. To address this, we used support vector machine (SVM) learning [15,16] to calculate the discrimination power of miR-941 with an SVM model. The result showed the area under the receiver operating curve (auROC) was 0.79 (Figure 1E), confirming that miR-941 expression alone is able to distinguish PsA patients from PsO or HCs.

### 2.3. Enhanced Osteoclast Activation and Bone Resorption in PsA Patients with Increased miR-941 Expression

**Figure 2:**
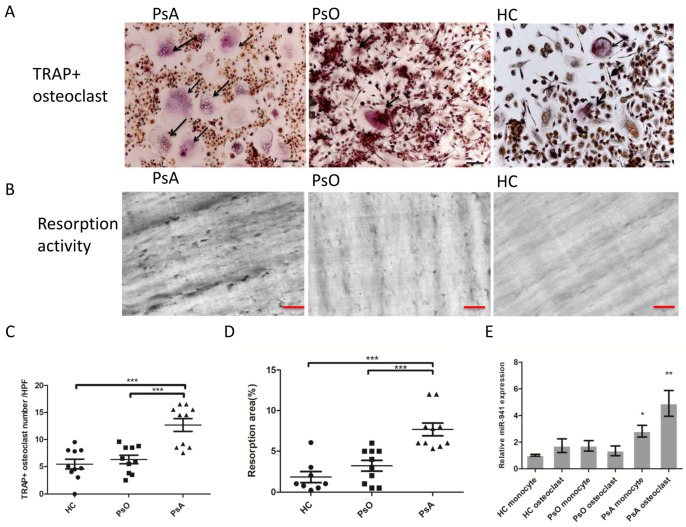
PsA patients with higher miR-941 expression demonstrated increased osteoclast differentiation potential and resorption activity derived from CD14+ monocytes. CD14+ monocytes from PsA patients, PsO patients, and HCs were cultured with macrophage colony-stimulating factor (M-CSF) 20 ng/mL for three days, followed by nine days in 100 ng/mL receptor activator of nuclear factor-κB ligand (RANKL) and 100 ng/mL tumor necrosis factor-α (TNF-α) for osteoclast differentiation. For evaluation of resorption activity, the osteoclasts were cultured on dentine slices. (**A**) At day 13, cells were stained with tartrate-resistant acid phosphatase (TRAP) to calculate the number of CD14+ monocyte-derived osteoclasts from PsA patients and NCs. Scale bar: 50 μm. (**B**) At day 13, resorption pits made by osteoclasts from psoriatic patients and NCs were recorded under a bright field microscope. Scale bar: 50 μm. (**C**) Osteoclast formation from CD14+ monocytes was compared between 10 HCs, 10 PsO patients, and 10 PsA patients. The number of osteoclasts was quantified among the three groups. (**D**) The eroded surface area on the dentine slices from PsA patients (*n* = 10), PsO patients (*n* = 10), and HCs (*n* = 10) were quantified using ImageJ and expressed as % of total dentine slice area. (**E**) The expression of miR-941 in monocytes and osteoclasts from PsA patients are higher than that from HCs and PsO patients using qRT-PCR. * *p* < 0.05; ** *p* < 0.01 and *** *p* < 0.001.

Given that miR-941 expression is elevated in CD14+ monocytes from PsA patients, we first examined whether these cells exhibited enhanced capacity to differentiate into osteoclasts and demonstrated enhanced resorption activity. The result showed that osteoclast formation was higher in CD14+ monocytes from PsA patients than that from PsO patients or HCs (12.7 ± 1.2, 6.3 ± 0.8, and 5.5 ± 0.9/per HPF from 10 PsA patients, 10 PsO patients, and 10 HCs, respectively, *p* < 0.001; Figure 2A,C). Regarding the bone resorption, the average percent area of resorption pits in dentine slices was also significantly higher in the differentiated osteoclasts from PsA patients than that from PsO patients or HCs (7.2 ± 2.0%, 2.9 ± 2.1%, 1.8 ± 1.8%/per HPF from average of 10 PsA samples, 10 PsO samples and 10 HC samples, respectively, *p* < 0.001; Figure 2B,D), indicating the enhanced osteoclast formation and active resorption activity from monocytes in PsA patients. To examine if miR-941 is important for osteoclastogenesis in PsA, we measured the expression of miR-941 in osteoclast from CD14+ monocytes of PsA, PsO patients and HCs. The expression of miR-941 in monocytes and osteoclasts from PsA patients are both higher than that from PsO patients (*p* < 0.05) or HCs (*p* < 0.05) (Figure 2E). These results suggest that miR-941 upregulation is in parallel with the elevated osteoclast differentiation potential in PsA patients.

### 2.4. miR-941 Inhibition Abolished the Osteoclast Activation and Functional Resportion in PsA Patients

**Figure 3:**
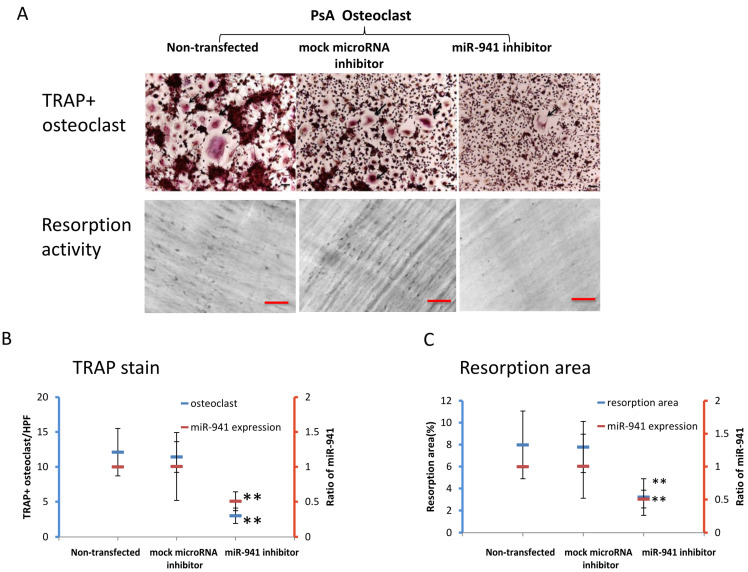
miR-941 inhibitor reversed osteoclast differentiation and bone resorption activity in patients with PsA. The CD14+ monocytes were obtained from eight PsA patients and were subsequently activated into osteoclasts with treatment of M-CSF for 72 h. Cells were either nontransfected or transfected with the control-microRNA inhibitor or miR-941 inhibitor followed by TNF-α and RANKL very three days for nine days to study their ability to form osteoclasts and their activity of bone resorption. At day 13, the number of osteoclast formations and percentage of resorption pits were measured. (**A**) The cells were stained with TRAP to calculate the number of osteoclasts among non-transection, negative control miRNA and miR-941 inhibitor transfection groups, Scale bar: 50 μm. For the evaluation of resorption activity, the resorption pits were recorded by a bright field microscope, Scale bar: 50 μm. (**B**) The number of osteoclasts was quantified among the three groups (blue). The relative expression of miR-941, as standardized to the level of non-transfected osteoclasts, was depicted as orange color. (**C**) The eroded surface areas on the dentine slice were quantified using ImageJ software as percentage of expressions in total areas (blue line). The expressions of miR-941 in the osteoclasts were measured by qRT-PCR to evaluate the effect of miR-941 inhibitors transfection. The relative expression of miR-941, as standardized to the level of non-transfected osteoclasts, was depicted as orange color. ** *p* < 0.01.

To address the direct involvement of miR-941 in the enhanced activation and functional resorption of osteoclasts derived from PsA patients, we examined whether miR-941 inhibition could reverse these effects. Peripheral CD14+ monocytes were obtained from 10 PsA patients and treated with M-CSF for 72 h. Cells were then divided into three treatment groups—one transfected with a control microRNA inhibitor (mock transfection control), one with a miR-941 inhibitor, and one without transfection. After transfection, cultures were treated with TNF-α and RANKL every three days for nine days to induce osteoclast formation. At day 13, the number of osteoclasts formed was significantly lower in the miR-941 inhibitor group (3.0 ± 1.1/HPF) than that in the non-transfected and mock transfected control groups (12.1 ± 3.4/HPF and 11.4 ± 2.2/HPF, respectively; *p* < 0.01) (Figure 3A,B). Similarly, resorption activity was significantly lower in the group transfected with miR-941 inhibitor (2.8 ± 1.2%) than that in the non-transfected and mock transfected control groups (7.2% ± 2.0% and 7.4% ± 2.0%, respectively; *p* < 0.01) (Figure 3A,C). The miR-941 inhibition abrogated the osteoclast development in PsA. The result also showed that microRNA-941 expression was reduced to 50% of baseline in the miR-941 inhibitor group as compared to that in the non-transfected and mock-transfected control miRNA groups (Figure 3B,C). These results indicated that the miR-941 inhibitor in CD14+ monocytes may reverse the active osteoclastogenesis and resorption activity in PsA patients.

### 2.5. MicroRNA-941 Inhibits WNT16 Expression in CD14+ Monocyte, and then Contributes to Active Osteoclastogenesis Independent of miR-146a-5p

**Figure 4:**
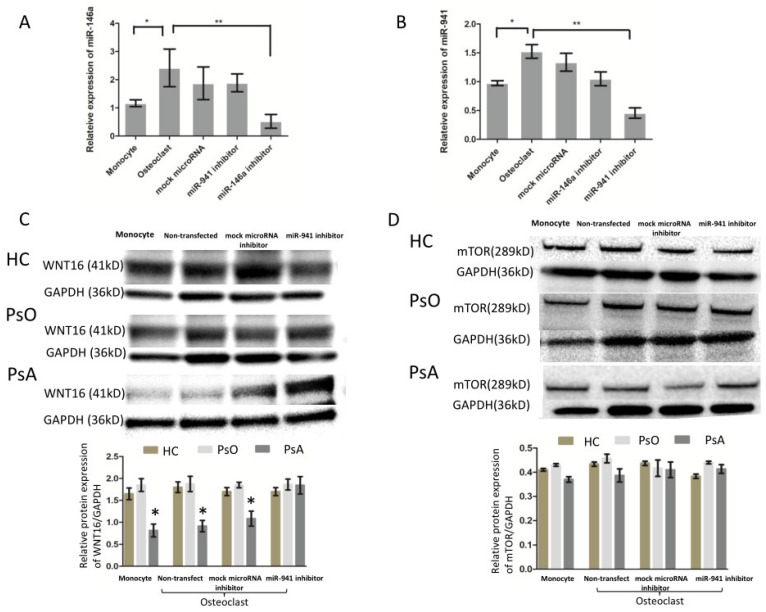
miR-941 inhibits osteoclastogenesis and negatively regulates WNT16 independent of miR-146a-5p. The CD14+ monocytes obtained from five PsA patients, five PsO patients, and five HCs were treated with medium only or subsequently activated into osteoclasts with treatment of M-CSF for 72 h. The cells with M-CSF treatment were either nontransfected or transfected with the control-microRNA inhibitor, miR-941 inhibitor or miR-146a-5p followed by TNF-α and RANKL every three days for nine days to study their ability to form osteoclasts and their activity of bone resorption. (**A**,**B**) At day 13, the expressions of miR-941 and miR-146a-5p in the monocytes and osteoclasts were measured by qRTPCR to evaluate the effect of miR-941 and miR-146a-5p inhibitors transfection. (**C**,**D**) The protein expression of WNT16 and mTOR, the target genes of miR-941, were measured by western blot. * *p* < 0.05 and ** *p* < 0.01.

Our previous study demonstrated that higher expression of miR-146a-5p in CD14+ monocytes of psoriatic arthritis than that in HCs. We then asked whether miR-941 and miR-146a-5p interact to activate osteoclastogenesis. Peripheral CD14+ monocytes were obtained from five PsA patients. Some cells were cultured with medium only while other cells were treated with M-CSF for 72 h. We then asked whether miR-941 regulated miR-146a-5p expression or vice versa. Therefore, four treatment groups were designed—one transfected with a control microRNA inhibitor (mock transfection control), one with a miR-941 inhibitor, one with miR-146a-5p inhibitor, and one without transfection. After transfection, cultures were treated with TNF-α and RANKL every three days for nine days to induce osteoclast formation. At day 13, expressions of miR-941 and miR-146a-5p in monocytes and monocyte-derived osteoclasts were measured. As anticipated, the results showed that the expressions of miR-941 and miR-146a-5p increased after osteoclastogenesis (Figure 4A,B). However, blocking miR-941 did not alter the expressions of miR-146a-5p. Conversely, blocking of miR-146a-5p did not alter the expressions of miR-941, either (Figure 4A,B). The results indicated that the two miRNA pathways may not directly interact with each other in the osteoclastogenesis of PsA. We next asked what regulatory pathways mediate the miR-941-induced osteoclastogenesis and bone resorption. Both hedgehog-signaling and insulin-signaling pathways were reported as the potential target genes of miR-941, including *SMO, SUFU, GLI, WNT16, IRS1, mTOR*, and *PPP1CA* [17]. Notably, specifically in the process of osteoclastrogenesis, Movérare-Skrtic et al. reported that WNT16 represses human osteoclastogenesis and prevents cortical bone fragility fractures through direct effects on osteoclast progenitors [18]. Hence, we examined whether miR-941 could negatively regulate WNT16 expression, leading to active osteoclastogenesis. To address that, we investigated if miR-941 inhibition could reverse the expression of WNT16 in CD14+ monocytes of PsA patients, PsO patients, and HCs. In addition, we also measured the expression of mTOR as an internal control in the same experimental setting. The result showed that WNT16 expression was significantly lower in monocytes, monocytes derived osteoclasts with/without mock microRNA transfection of PsA patients compared to those from PsO patients or HCs (*p* < 0.05). The expression of WNT16 in osteoclast with miR-941 inhibitor from PsA patients is similar to that from PsO patients or HCs (Figure 4C). In contrast, the expression of mTOR did not change with or without miR-941 inhibition (Figure 4D).

### 2.6. The Enhanced miR-941 Expression was Reduced in CD14+ Cells from PsA Patients after Successful Biologics Treatment

**Figure 5:**
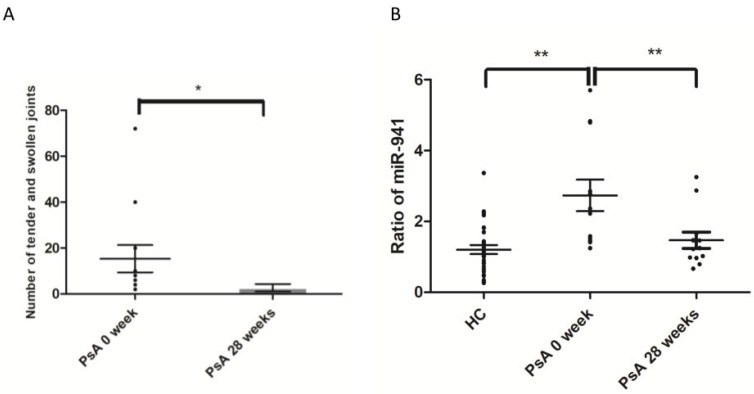
Reduced miR-941 expression in CD14+ monocytes from PsA patients after successful biologics treatment. The expression levels of miR-941 were measured in CD14+ monocytes from HCs and PsA patients before and after successful biologic treatment. (**A**) The total number of tender and swollen joints in PsA patients were measured before and 28 weeks after biologics treatment. (**B**) The expression levels of miR-941 were measured in CD14+ monocytes from 31 NCs and 12 PsA patients before and after 28 weeks of biologics treatment using qRT-PCR. * *p* < 0.05; ** *p* < 0.01.

To address whether the expression of miR-941 from circulating monocytes might serve as a potential biomarker, we examined whether the increased miR-941 expression in CD14+ monocytes from PsA patients was reduced after successful treatment. Among the 12 PsA patients that met the ACR20 achievement after 28 weeks of biologics treatment (etanercept, adalimumab, secukinumab, or ustekinumab) (Figure 5A), the enhanced expression of miR-941 in CD14+ monocytes returned to the level of NCs (Figure 5B). 

## 3. Discussion

Our study investigated the overall expression of miRNAs from CD14+ monocytes of PsA patients. Expression of miR-941 was higher in CD14+ monocytes from PsA patients than that from PsO patients and HCs, and CD14+ monocytes from PsA patients tend to exhibit enhanced osteoclast differentiation potential and resorption activity. Further, miR-941 inhibition resumed the elevated osteoclast formation potential and resorption activity of PsA monocytes with a reciprocal upregulation of WNT16. The expression of miR-941 in CD14+ monocytes of PsA patients correlated with disease severity.

Pathological bone resorption in PsA results from increased numbers of osteoclast precursors [8]. Early diagnosis of PsA has been hindered by the lack of reliable biomarkers. Our study identifies miRNA-941 in CD14+ monocytes as an easily accessible marker for prompt computer-aided diagnosis of PsA (with an auROC value 0.79 by SVM model). In addition, we demonstrate that the increased miR-941 expression in CD14+ monocytes from PsA patients enhances osteoclast formation potential and active resorption activity. Our previous study reported that miR-146a-5p expression in CD14+ monocytes of PsA patients correlates with clinical efficacy and inducts osteoclast activation and bone resorption [11]. In this study, the overall microRNAs were measured using NGS study. The result showed that the increased expression of miR-941 from CD14+ monocytes could be used for detection of PsA. In addition, miR-941 and miR-146a-5p did not interact to activate osteoclastogenesis. In other disease, miR-941 has been indicated to be a severity marker. For example, in acute coronary syndrome, miR-941 expression was reported to be increased in the plasma of the patients [19]. Duttagupta et al. identify that miR-941 is associated with ulcerative colitis [20]. 

The pro-inflammatory cytokines, including TNF-α, IL-17, IL-33, and osteopontin, through activation of RANKL, induce osteoclast differentiation and activation in PsA [21,22,23,24]. Biologics selectively targeting these specific cytokines (TNF-α, IL-17 and IL-12/23) and/or intracellular signaling pathways effectively inhibit osteoclastogenesis. However, more than 40% of PsA patients show only a partial response or fail to respond to current biologics [25]. On the other hand, both enhanced osteoclast differentiation and bone resorption could be reversed by miR-941 inhibition in CD14+ monocytes from PsA patients in this study (*n* = 10), indicating that miR-941 may serve as both a potential biomarker and treatment target for PsA. 

WNT16 is a strong anti-resorptive soluble factor acting on osteoclast precursors [26]. It can significantly inhibit RANKL-induced osteoclastogenesis of human CD14+ peripheral blood monocytes, indicating a direct action of WNT16 on osteoclast precursors. Reduced osteoclast formation by WNT16 was associated with a time- and dose-dependent blunted mRNA expression of osteoclast functional genes [18,27]. Our results provide miR-941 inhibitor increased the expression of WNT16 and inhibit osteoclastogenesis, indicating the potential involvement of miR-941-WNT in the active osteoclastogenesis in patients with PsA.

This study has several limitations. First, the case number was small. The results may be validated by large-scale studies to identify potential confounders. Second, the PsA patients recruited may have different intrinsic co-morbidities that also alter miRNA expression (e.g., diabetes mellitus, cerebrovascular disease, hypertension, etc.), making these confounders could potentially interfere the results. Third, the expression level of miR-941 in CD14+ monocytes from PsA patients before and after conventional synthetic disease-modifying antirheumatic drugs was not investigated. Fourth, although there was a reciprocal change of miR-941 and WNT16 expression in this study, whether WNT16 could be a direct target for miR-941 may require a luciferase assay. 

In conclusion, the elevated expression of miR-941 in CD14+ monocytes from PsA patients enhances osteoclast activation and bone resorption activity through WNT16 repression. The activation of miR-941 and reciprocal changes of WNT16 in osteoclasts contribute to bone destruction in PsA.

## 4. Materials and Methods 

### 4.1. Study Subjects 

This study was conducted in accordance with the Declaration of Helsinki and was approved by the institutional review board of Chang-Gung Memorial Hospital, Taiwan (104-9618A3, 01/08/2016). Signed informed consent was obtained from all patients. 40 PsA patients and 40 PsO patients were diagnosed by both dermatologists and rheumatologists. All patients of the PsA group fulfilled the CASPAR criteria. 40 healthy adults were included as a control group (HC). The absence of psoriatic lesions and inflammatory joint were examined thoroughly in HC group. The Psoriasis Area and Severity Index (PASI) score, comorbidities of arthritis, presence of uveitis and treatment regimens were recorded. Peripheral blood from all participants was acquired at baseline and from patients after 28 weeks of standard biologics treatment (etanercept, adalimumab, secukinumab, or ustekinumab).

### 4.2. Isolation and Culture of Peripheral Monocytes

Monocytes were isolated directly from PBMCs using CD14+ MicroBeads (Miltenyi Biotec, Auburn, CA, USA) according to the manufacturer’s instructions. The purity of the CD14+ cells after the selection is above 95% using flow cytometry showed according to our previous study [28].

### 4.3. MicroRNA Expression Profiling in CD14+ Monocytes by Next-Generation Sequencing (NGS)

RNA of CD14+ monocytes was extracted using Direct-zol™ RNA Kits (Zymo Research, Irvine, CA, USA) according to the manufacturer’s protocol and measured quantitatively using Agilent Bioanalyzer 2100 O (Agilent, Santa Clara, CA, USA). Only samples with RNA integrity number (RIN) ≥ 8.0 were then used for the TruSeq Small RNA Preparation protocol (Illumina, San Diego, CA, USA). The prepared amplicons were sequenced with a V3 150-cycle sequencing reagent on the MiSeq system (Illumina, San Diego, CA, USA) to generate 51-nt single-end reads. The generated NGS data were first analyzed with miRSeq [29], a toolkit for sequencing quality evaluation and miRNA quantification (in transcripts per million, TPM).

### 4.4. Osteoclast Formation

Then, 3 × 10^5^ purified human CD14+ monocytes were seeded in 24-well plates containing a-MEM with FBS (10%, *v*/*v*; Invitrogen, Waltham, MA, USA) and M-CSF (20 ng/mL; PeproTech, Rocky Hill, NJ, USA) for three days. RANKL (100 ng/mL; PeproTech, Rocky Hill, NJ, USA) and TNF-α (100 ng/mL; PeproTech, Rocky Hill, NJ, USA) were added to induce osteoclast differentiation [11]. The osteoclasts were stained with tartrate-resistant acid phosphatase (TRAP) at day 13 using the Acid Phosphate Leukocyte Kit (Sigma, St. Louis, MO, USA) according to the manufacturer’s instructions. TRAP-stained cells containing three or more nuclei were defined as osteoclasts [30]. We measured the number of osteoclasts from four randomly selected higher power fields (HPF) (200×) per quadrant of the well. Four HPF were chosen from each quadrant. The number of osteoclasts was counted from the average value from 16 HPFs. 

### 4.5. Bone Resorption Assay

Here, 5 × 10^4^ purified human CD14+ monocytes were seeded on dentine slices (IDS, Gaithersburg, MD, USA) in 96-well plates containing a-MEM with 10% FBS and M-CSF (20 ng/mL) for 72 h. The cells were subsequently incubated with RANKL (100 ng/mL) and TNF-α (100 ng/mL) to induce osteoclast differentiation. At day 13, dentine slices were imaged using a bright filed microscope (Leica DM2500, Wetzlar, Germany). The Bone resorption pits were calculated using ImageJ software (NIH, Bethesda, MD, USA) from four randomly selected HPFs.

### 4.6. Transient Transfection of miR-941 Inhibitors

Isolated CD14+ monocyte was cultured in a-MEM with 10% FBS and M-CSF for 72 h in 96-well plates on dentine slices. Cells were then transfected with 10 nM hsa-miR-941 hairpin inhibitor or 10 nM miRNA hairpin inhibitor as a negative control (Dharmacon, Lafayette, CO, USA) using lipofectamine 3000 for 6 h based on manufacturer’s instructions (Invitrogen, Carlsbad, CA, USA). The sequences of miR-941 inhibitor and negative control miRNA are GUGGGCCGACACACGUGUACACG and UCACAACCUCCUAGAAAGAGUAGA. The transfection efficiency was measured by qRT-PCR.

### 4.7. Quantitative Real-Time Reverse Transcription Polymerase Chain Reaction (qRT-PCR) Analysis

The complementary DNAs (cDNAs) were obtained from RNA samples (100 ng per run) using a TaqMan MicroRNA Reverse Transcription kit (Applied Biosystems; Thermo Fisher Scientific, Inc, Carlsbad, CA, USA) according to the manufacturer’s protocol. Expressions of miR-941 (Assay ID. 002183) and miR-146b-5p (Assay ID. 001097) were examined using TaqMan microRNA assays (Applied Biosystems; Thermo Fisher Scientific, Inc, Carlsbad, CA, USA). The following primers of miRNAs were used: hsa-miR-941, UGAGAACUGAAUUCCAUGGGUU and hsa-miR-146b-5p, UGAGAACUGAAUUCCAUAGGCU. Quantitative RT-PCR was performed on an Applied Biosystems QuantStudio 7 Flex system. Target gene expression levels were normalized to U6 (Assay ID. 4427975). We quantified the PCR values using a 2-ΔΔCt method with U6 as an internal control by adopting the method from a previous study [31]. 

### 4.8. Western Blot Analysis

Total proteins from monocytes and monocytes-derived osteoclasts from patients with PsA were separated on a 10% SDS polyacrylamide gel and the proteins transferred to a PVDF membrane (Merck Millipore, Darmstadt, Germany). Non-specific binding sites were blocked with 5% BSA for one hour at room temperature. Membranes were subsequently incubated overnight at 4 °C with rabbit anti-WNT16 (Invitrogen, Carlsbad, CA, USA), rabbit anti-mTOR (Invitrogen, Carlsbad, CA, USA), and mouse anti-GAPDH (Merck Millipore, Darmstadt, Germany) antibodies conjugated with horseradish peroxidase. Band densities were quantified using ImageJ software (NIH, Bethesda, MD, USA).

### 4.9. Statistical Analysis

Age, sex, PASI score, number of tender or swollen joints, disease duration, treatment regimen, miRNA expression level, number of osteoclasts formation, and resorption area were compared among groups by chi-square, *t*-test, One-way ANOVA, or logistic regression based on the data normality. Receiver-operator characteristic analysis (ROC) and area under the curve (AUC) were used to assess whether expressions of miRNAs in CD14+ monocytes, could distinguish between PsA samples and PsO samples or healthy controls. A *p*-value less than 0.05 was considered statistically significant for all tests.

## Figures and Tables

**Table 1:** Demographics of psoriatic arthritis patients (PsA), psoriatic patients without arthritis (PsO), and healthy controls (HCs).

	PsA (*n* = 40)	PsO (*n* = 40)	HC (*n* = 40)
Age (years)	47.6 ± 12.2	43.8 ± 13.3	44.1 ± 12.4
Female/Male	12/28	9/31	11/29
Weight (kg)	72.4 ± 15.5	73.1 ± 14.1	70.0 ± 10.0
Psoriasis duration (years)	14.9 ± 7.4	15.8 ± 7.4	
Psoriatic arthritis duration (years)	7.9 ± 7.2		
Skin PASI *	14.2 ± 9.1	15.9 ± 5.3	
Peripheral arthritis no. (%)	40 (100)		
Peripheral and axil arthritis no. (%)	14 (35)		
Dactylitis no. (%)	14 (35)		
Enthesitis no. (%)	18 (45)		
No. of tender-joints (78 joints)	7.5 ± 7.0		
No. of swollen-joint (76 joints)	6.7 ± 6.9		
Uveitis no. (%)	2 (5)		
Previous drug usage: Anti-TNF, anti-IL12/23 or anti-IL17 biologics. no. (%)	4 (10)	4 (10)	
Methotrexate no. (%)	31 (77.5)	33 (82.5)	
Leflunomide no. (%)	10 (25)	0	
NSAID no. (%)	38 (95)	0	

* PASI: Psoriasis Area and Severity Index.
